# Challenges in the Development of Intravenous Neurokinin‐1 Receptor Antagonists: Results of a Safety and Pharmacokinetics Dose‐Finding, Phase 1 Study of Intravenous Fosnetupitant

**DOI:** 10.1002/cpdd.1183

**Published:** 2022-10-20

**Authors:** Timothy Tyler, Armin Schultz, Alessio Venturini, Claudio Giuliano, Alberto Bernareggi, Riccardo Spezia, Daniel Voisin, Valentino Stella

**Affiliations:** ^1^ Comprehensive Cancer Center Desert Regional Medical Center Palm Springs California USA; ^2^ CRS Clinical Research Services Mannheim GmbH Mannheim Germany; ^3^ Helsinn Healthcare SA Lugano/Pazzallo Switzerland; ^4^ Pharmaceutical Chemistry The University of Kansas Lawrence Kansas USA

**Keywords:** chemotherapy‐induced nausea and vomiting, fosnetupitant, 5‐hydroxytryptamine‐3 receptor antagonists, injection site reactions, intravenous formulation, netupitant, neurokinin‐1 receptor antagonists, palonosetron, prodrug

## Abstract

Oral NEPA is the fixed‐combination antiemetic comprising netupitant (neurokinin‐1 receptor antagonist [NK_1_RA]) and palonosetron (5‐hydroxytryptamine‐3 receptor antagonist [5‐HT_3_ RA]). Intravenous (IV) NEPA, containing fosnetupitant, a water‐soluble N‐phosphoryloxymethyl prodrug of netupitant, has been developed. Fosnetupitant does not require excipients or solubility enhancers often used to increase IV NK_1_RA water solubility, preventing the occurrence of hypersensitivity and infusion‐site reactions associated with these products. In this phase 1 study, subjects received a 30‐minute placebo or fosnetupitant (17.6–353 mg) infusion and an oral NEPA or placebo capsule, with 2‐sequence crossover treatment for fosnetupitant 118‐ to 353‐mg dose cohorts. IV fosnetupitant safety and pharmacokinetics were evaluated, and its equivalence to an oral netupitant 300‐mg dose was defined. Overall, 158 healthy volunteers were enrolled. All adverse events (AEs) were mild or moderate in intensity. Doppler‐identified infusion‐site asymptomatic thrombosis occurred in 5.4% (fosnetupitant) and 1.2% (oral NEPA) of subjects. The frequency or number of treatment‐related AEs did not increase with ascending fosnetupitant doses. The most common treatment‐related AEs were headache (fosnetupitant, 8.1%; oral NEPA, 12.7%) and constipation (fosnetupitant, 1.4%; oral NEPA, 7.5%). A fosnetupitant 235‐mg dose was equivalent, in terms of netupitant exposure, to 300‐mg oral netupitant. The safety profile of a single fosnetupitant 235‐mg infusion was similar to that of single‐dose oral NEPA.

Chemotherapy‐induced nausea and vomiting (CINV) is a common and distressing side effect associated with many anticancer treatments that remains a significant clinical challenge. As uncontrolled CINV may prevent anticancer treatment completion and negatively impact the patient's quality of life,^1^, ^2^ steps to prevent and manage its occurrence are essential for patient health. The current standard of care in the highly emetogenic chemotherapy (HEC; including anthracycline‐cyclophosphamide [AC]) and carboplatin (area under the concentration‐time curve [AUC] ≥4 mg/mL/min) setting is a combination of neurokinin‐1 receptor antagonists (NK_1_RAs), 5‐hydroxytryptamine‐3 receptor antagonists (5‐HT_3_RAs), and the glucocorticosteroid dexamethasone.

While 5‐HT_3_RAs were initially developed for intravenous (IV) use, NK_1_RAs originated as orally administered agents, and in later years there has been a drive to produce IV formulations to increase administration convenience. However, with injectable use, their excipients and the physicochemical properties of the active agents themselves may be associated with injection site reactions (ISRs), which manifest as pruritus, swelling, erythema, and pain around the injection site, or other side effects. Such adverse events (AEs) are associated with a number of causes, including chemical, microbiologic, and hemolytic factors, as well as mechanically induced irritation around the puncture site.

Given the concerns about the ISRs associated with various NK_1_RA IV formulations, there is a need to optimize dosing schedules to minimize the risk of such reactions. The present study aimed to evaluate the safety of ascending doses of IV fosnetupitant, a novel prodrug of netupitant (Figure 1).

**Figure 1:**
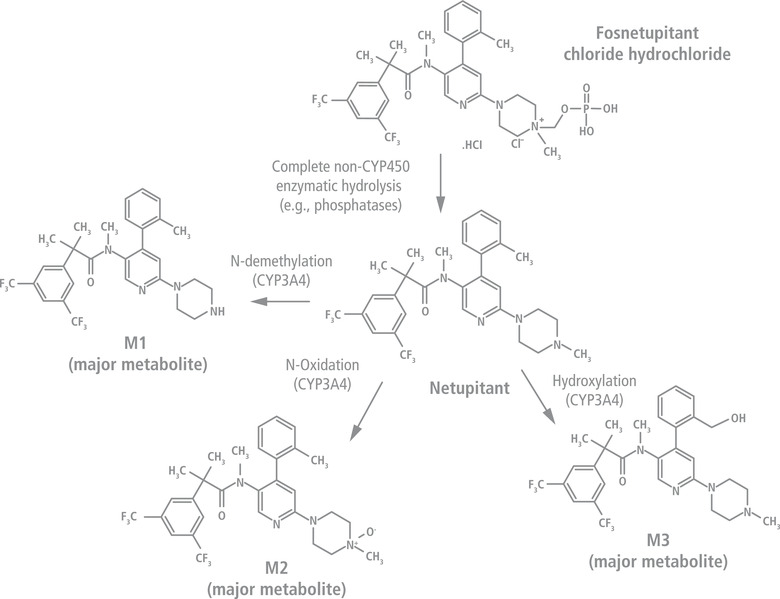
Chemical structures of fosnetupitant, netupitant, and netupitant metabolites M1, M2, and M3. CYP, cytochrome P450.

Certain excipients, such as synthetic nonionic surfactants that are used to improve the solubility of poorly water‐soluble drugs, are not inert and have biologic activity of their own.^3^ One example is polysorbate 80, composed of fatty acid esters and polyoxyethylene sorbitan,^4^ which is used in the fosaprepitant formulation^5^ and has been associated with hypersensitivity reactions.^6^ The IV formulation of rolapitant, which uses the synthetic surfactant polyoxyl 15 hydroxystearate,^7^, ^8^ has also been shown to cause serious AEs including anaphylaxis and anaphylactic shock.^9^

Complications may also result from phase separation, where crystals or oil droplets of the drug are formed upon mixing. If the precipitation of the solubilized drug is crystalline, it may cause physical damage such as cellular abrasion from the interaction of the particles with the vein wall.^10^ The introduction of particulate matter during injections^10^ and the spontaneous crystallization during administration that can occur if solubility limits are approached during dilution^11^ may cause mechanical and chemical effects. These may result in phlebitis that can cause potentially fatal thrombus formation. Hemolysis is another problem observed after IV infusion, either from hypotonicity or from the action of the drug or formulation components on cell membranes.

Infusions of all NK_1_RAs are associated with ISRs, including phlebitis/thrombophlebitis and hypersensitivity reactions (such as anaphylaxis).^12^, ^13^, ^14^ This is mainly due to the chemical and physical properties of the drugs. NK_1_RAs are amphiphilic molecules that tend to have low solubility in water,^8^, ^15^, ^16^, ^17^, ^18^ can interfere with the lipid matrix of cell membranes, and are potentially hemolytic,^19^ which may ultimately trigger ISRs. Two different strategies have been undertaken to develop safer formulations with reduced potential for developing ISRs. One approach has been to develop a formulation that is able to maintain the solubility of the NK_1_RA, thus avoiding its irritant features, and the other is the creation of a prodrug with higher water solubility than the parent drug.

## Formulation Approach

The novel approach to increase solubility with a tolerable formulation was used in the development of IV rolapitant, the early IV netupitant and aprepitant preparations, and HTX‐019 (aprepitant IV emulsion). The rolapitant IV emulsion, which contained polyoxyl 15 hydroxystearate, was found to cause anaphylaxis, anaphylactic shock, and other serious hypersensitivity reactions^20^ and was subsequently withdrawn from the market in the European Union, and a safety warning was issued in the United States.^9^ Both the first IV netupitant and aprepitant formulations were also discontinued in early development due to tolerability issues, predominantly thrombophlebitis. Infusion of IV netupitant, containing polysorbate 80, resulted in mild to moderate infusion‐site thrombosis (thrombophlebitis) in 4 (67%) trial participants treated at the subtherapeutic dose of 30 mg. In all cases, the thrombophlebitis affected the dosing vein and was considered probably related to the trial treatment (Helsinn, data on file). The incidence, duration, and intensity of the ISRs were correlated to the dose of the drug, and, most likely, to the drug concentration at the infusion site and the peak serum concentration. The first IV aprepitant formulation was also discontinued in early development due to the incidence of induration, tenderness, infusion‐site pain, and swelling.^21^ The second IV aprepitant formulation, HTX‐019, was developed as a polysorbate 80– and surfactant‐free injectable emulsion,^22^ and evidence suggests this is more tolerable. However, to increase solubility, the emulsion contains ethanol and several potentially allergenic ingredients, such as egg lecithin and soybean oil. In a bioequivalence study of fosaprepitant versus IV HTX‐019, infusion‐site pain was reported in just 1% of subjects, although it is noteworthy that AEs were monitored for only 1 hour following infusion.^22^

## Prodrug Approach

Intravenously administered prodrugs deliver the active drugs in the bloodstream, following their cleavage in vivo. Consequently, ISRs that may occur as a result of excessive concentrations of the active drug at the injection site should be minimized, provided the prodrug itself is safe. Physical or chemical modifications are also used to improve the tolerability of the active drug, often by increasing its solubility especially at physiologic pH, which further reduces the incidence of ISRs. Several antiemetics have been developed using this strategy. Fosaprepitant (dimeglumine) is a water‐soluble N‐phosphoryl prodrug of aprepitant that is rapidly converted in vivo to aprepitant. Its formulation, however, includes polysorbate 80.^5^ Infusion of fosaprepitant has been associated with ISRs such as pain, irritation, puncture‐site pain, induration, swelling, and thrombophlebitis; vasculitis and necrosis were also reported following infusion with concomitant vesicant chemotherapy.^23^ Most importantly, serious systemic hypersensitivity reactions such as anaphylaxis have been reported either during or shortly after infusion and emphasize the need for specific precautions, especially for patients with known allergies to medications containing polysorbate 80.^5^, ^12^, ^24^ Fosnetupitant is a novel prodrug of netupitant (Figure 1) that, following infusion, is converted in vivo to its active form, netupitant.^25^ The IV formulation of fosnetupitant as part of IV NEPA (fosnetupitant 235 mg/palonosetron 0.25 mg) was found to be safe and tolerable with no ISRs reported.^25^, ^26^, ^27^ The fosnetupitant formulation does not require surfactants (such as polysorbate 80 or polyoxyl 15 hydroxystearate), emulsifiers, or solubility enhancers to achieve complete solubility. Moreover, this formulation does not contain alcohol or allergic components, such as soya or egg derivatives.^27^

Previous studies have shown that the oral netupitant 300‐mg dose is absorbed rapidly, with maximum plasma concentration (C_max_) ranging from 99 to 517 μg/L reached at 2–5.5 hours after administration. Netupitant undergoes extensive metabolism, primarily by cytochrome P450 (CYP) 3A4 enzymes,^28^ leading to formation of 3 major pharmacologically active metabolites—M1, M2, and M3—which are detectable 1 hour after administration.^29^, ^30^ In preclinical studies, M3 showed pharmacologic potency similar to netupitant, whereas M1 and M2 potency was lower than the parent drug (Helsinn, data on file). The elimination half‐life (t_1/2_) ranges from 75 to 101 hours, with elimination of netupitant and its metabolites occurring mainly via the hepatic/biliary route.^29^, ^30^ Pharmacodynamic studies with NK_1_RAs have shown that >90% NK_1_ receptor occupancy in the striatum is correlated with clinical activity, and this receptor occupancy threshold is used as a surrogate marker of NK_1_RA activity.^31^ A single oral 300‐mg netupitant dose results in 87%–98% and 75%–77% NK_1_ receptor occupancy in the striatum at 6 and 96 hours after administration, respectively.^29^ Furthermore, pharmacologic models predict maximal receptor occupancy achieved by 3 hours in all brain regions and 75% occupancy in the striatum at 120 hours following administration.^32^ Netupitant and its metabolite M1 are inhibitors of CYP3A4 and in vivo may increase the exposure of CYP3A4 substrate drugs.^33^ Because NEPA is administered with dexamethasone (a known CYP3A4 substrate) for antiemetic prophylaxis, the dose of dexamethasone is reduced in NEPA‐based regimens.^34^, ^35^, ^36^

Fosnetupitant is rapidly and completely hydrolyzed to netupitant through the action of phosphatases and esterases, the primary elimination route (Helsinn, data on file), and then is metabolized to M1, M2, and M3.^30^ In patients with cancer, fosnetupitant C_max_ is reached at the end of the 30‐minute infusion of 235‐mg fosnetupitant, with <1% of the prodrug being detectable 30 minutes after the end of infusion.^25^ The t_1/2_ of netupitant was 144 hours, with M1, M2, and M3 metabolites being detectable at 2 hours after infusion.^25^ In the present study, to evaluate the local tolerability and safety of ascending doses of IV fosnetupitant, the presence of any asymptomatic (hence difficult to detect) local adverse reactions was assessed via color Doppler ultrasound scanning of the infusion and contralateral veins. A secondary objective was to investigate the pharmacokinetics (PK) of fosnetupitant and define the dose of fosnetupitant that is equivalent to the oral netupitant 300‐mg dose in terms of netupitant exposure. This information was needed for performing further development activities on the IV NEPA formulation. Herein, we present the results of the phase 1, dose‐escalating study with fosnetupitant in healthy adult volunteers.

## Methods

### Study Design and Treatment

This was a single‐center, randomized, 2‐period, 2‐sequence crossover, phase 1 study in healthy male and female volunteers (EudraCT 2012‐003407‐35) performed in different parts, where subjects received IV fosnetupitant or oral NEPA (Supplemental Figure). Part I was a parallel‐group single‐ascending‐dose (SAD) part with a crossover extension for cohorts treated with IV fosnetupitant doses ≥118 mg. The subjects were randomized 4:1 to IV fosnetupitant or oral NEPA, and 10 volunteers were assigned to each of the 8 dose cohorts planned, ranging from 17.6 mg to 353 mg IV fosnetupitant (Supplemental Figure). Part II was a pilot crossover in 20 subjects for the 176‐ and 235‐mg dose cohorts of IV fosnetupitant, selected on the basis of part I PK data. All subjects were randomized 1:1 to initially receive IV fosnetupitant or oral NEPA following a crossover design. Part III was the final crossover part for 19 subjects in the 212‐mg IV fosnetupitant cohort and 20 subjects in the 259‐mg cohort (90% and 110%, respectively, of the identified “target dose” [235 mg]). Subjects were randomized 1:1 to initially receive IV fosnetupitant or oral NEPA following a crossover design. The 2 doses for the final crossover part were selected on the basis of PK data from parts I and II and estimation of the relative bioavailability of IV fosnetupitant compared with oral NEPA, which was used to identify the IV fosnetupitant dose (target dose) that yields equivalent netupitant exposure to that obtained from netupitant 300 mg in oral NEPA. Finally, all 3 parts contributed to the final estimation. All parts were performed under double‐blind (within dose cohorts), double‐dummy conditions.

IV fosnetupitant or placebo was infused over 30 minutes immediately following the intake of a placebo or oral NEPA capsule on day 1. Each treatment period was separated by a washout phase of at least 4 weeks. An independent drug safety monitoring board (DSMB) participated in the dose‐escalating process by evaluating the clinical significance of the safety data collected from each dose cohort in the SAD part. The DSMB also assessed safety data at the end of the pilot crossover and final crossover parts. The primary objective of this study was to assess the safety of fosnetupitant administered as a single IV infusion. The secondary objective was to identify the IV fosnetupitant dose that is equivalent in terms of netupitant exposure to the oral netupitant 300‐mg dose present in oral NEPA; this dose was to be selected for further development activities.

Subjects provided signed informed consent before enrollment, in compliance with the Declaration of Helsinki. The study was conducted at CRS Clinical Research Services Mannheim GmbH in accordance with the Declaration of Helsinki, the German Drug Law (Arzneimittelgesetz), the German Good Clinical Practice decree, and the Note for Guidance on Good Clinical Practice. The study was approved by the independent ethics committee of the “Landesärztekammer Baden‐Württemberg” and the competent authority (Bundesinstitut für Arzneimittel und Medizinprodukte) on November 6, 2012.

### Eligibility Criteria

Male and female subjects aged 18–45 years, with a body weight ≥50 kg, body mass index between 18.5 and 29 kg/m^2^, and who were in general good health were included in the study.

### Safety Assessments

AEs were assessed and coded according to the Medical Dictionary for Regulatory Activities. AE severity and relationship to the study drug were assessed by both the investigator and the DSMB according to the Common Terminology Criteria for Adverse Events version 4.0 and other indications provided in the study protocol. Additional safety assessments comprised clinical laboratory tests (including specific laboratory parameters for the local tolerability evaluation), vital signs, 12‐lead electrocardiogram, physical examination, and Doppler ultrasound scanning of the infusion and contralateral veins. Color Doppler ultrasound scanning was used to determine possible damage to the infusion and contralateral veins. As predefined in the study protocol, scans were obtained with higher frequency in the SAD/SAD crossover parts of the study and were assessed after each dose cohort by the investigator and DSMB.

### Pharmacokinetic Assessments

Blood samples for PK analyses were collected on day 1 before dosing; at 15 (middle of infusion), 30 (end of infusion), 40, and 50 minutes; and at 1, 1.25, 1.5, 2, 3, 4, 5, 6, 8, 12, 24, 48, 72, 96, and 120 hours from the start of infusion. The analytic methods used for the detection of fosnetupitant, netupitant, and netupitant metabolites M1, M2, and M3 are described in the Supplemental Information. PK parameters, calculated from plasma concentrations of fosnetupitant, netupitant, M1, M2, and M3 by noncompartmental analysis, included C_max_, AUC from time 0 to the time of last measurable concentration (AUC_0‐last_), AUC from time 0 to infinity (AUC_0‐inf_), time to reach maximum concentration of drug in plasma, and t_1/2_. Calculations were performed using the actual sampling times.

### Statistical Methods

Plasma concentrations and PK parameters were analyzed in the restricted PK analysis set, including all randomized subjects in the SAD and crossover parts who received the whole dosage of the active treatment (IV fosnetupitant or oral NEPA in the SAD part and IV fosnetupitant and oral NEPA in the crossover parts) and for whom the AUC_0‐inf_ for the corresponding periods could be obtained.

The equivalence of a 235‐mg IV fosnetupitant dose (test [T]) and the reference 300‐mg oral netupitant dose administered as oral NEPA (R) was assessed by comparing the systemic exposure of netupitant following administration of the respective doses. The T/R AUC_0‐last_ and AUC_0‐inf_ geometric least squares means (GLSM) ratios were calculated along with their 90%CI. Bioequivalence was determined by demonstrating that the 90%CI of the T/R AUC_0‐last_ and AUC_0‐inf_ GLSM ratios were within the 80.0%–125.0% acceptance interval for bioequivalence.^37^

Descriptive statistics were used to summarize PK parameters of fosnetupitant, netupitant, and netupitant metabolites by treatment group, the measured variables, and the derived safety parameters. Descriptive statistics were used for the frequency of treatment‐emergent AEs (TEAEs). The safety analysis set consisted of all subjects treated with at least part of the IV infusion treatment or at least 1 dose of the oral treatment and who had at least 1 safety assessment after treatment.

## Results

### Subjects

In total, 160 subjects were randomized, of whom 130 were randomly assigned to crossover treatment cohorts; 158 subjects constituted the safety analysis set and 153 the restricted PK analysis set. The Supplemental Figure illustrates the subject disposition in the different study parts. The main demographics and baseline characteristics are shown in Table 1. The majority of subjects were men (59.5%) and White (92.4%), the median age was 32.5 years, and the mean body mass index was 24.6 kg/m^2^.

**Table 1:** Subject Demographics and Baseline Characteristics—Safety Analysis Set

Parameter	Overall IV (N = 148)	Overall Oral (N = 134)	Total (N = 158)
Sex, *n* (%)			
Female	43^a^	21^b^	64 (40.5)
Male	60^a^	34^b^	94 (59.5)
Age, y			
Mean (SD)	32.5 (7.7)	32.2 (7.8)	32.5 (7.7)
Median (range)	33.0 (18–45)	32.0 (18–45)	32.5 (18–45)
Race, *n* (%)			
White	97^a^	49^b^	146 (92.4)
Black	3^a^	3^b^	6 (3.8)
Asian	3^a^	1^b^	4 (2.5)
Other	0^a^	2^b^	2 (1.3)
Weight, kg			
Mean (SD)	74.8 (12.2)	76.0 (12.5)	75.1 (12.4)
Median (range)	76.7 (50.3–113.2)	78.6 (50.3–113.2)	77.5 (50.3–113.2)
Height, cm			
Mean (SD)	174.1 (9.2)	174.7 (9.4)	174.3 (9.2)
Median (range)	174.5 (152–198)	176.0 (152–198)	175.0 (152–198)
BMI, kg/m^2^			
Mean (SD)	24.6 (2.6)	24.8 (2.6)	24.6 (2.6)
Median (range)	24.8 (18.8–29.4^c^)	25.2 (19.3–29.4^c^)	24.8 (18.8–29.4^c^)

BMI, body mass index; IV, intravenous infusion of 17.6 to 353 mg fosnetupitant and administration of placebo capsule; Oral, oral NEPA (netupitant 300 mg/palonosetron 0.5 mg) administration and placebo infusion; SD, standard deviation.

^a^ Group of subjects that received: IV treatment – oral treatment or IV treatment, depending on cohort (n = 103).

^b^ Group of subjects that received: oral treatment – IV treatment or oral treatment, depending on cohort (n = 55).

^c^ Acceptance window for upper limit of BMI: 29.0–29.4 kg/m^2^.

### Safety

TEAEs occurring in >1% of the 158 subjects are summarized in Table 2. In total, 169 TEAEs and 108 treatment‐related AEs (TRAEs), all of mild or moderate intensity, were reported in 80 (50.6%) and 59 (37.3%) subjects, respectively. Single ascending doses of IV fosnetupitant were applied in this study. The frequency of subjects with TRAEs or the number of TRAEs did not increase with rising fosnetupitant dose (17.6 mg: 2 [25%] subjects, 2 events; 29.4 mg: 3 [38%] subjects, 3 events; 59 mg: 1 [13%] subject, 1 event; 118 mg: 2 [20%] subjects, 7 events; 176 mg: 6 [21%] subjects, 8 events; 212 mg: 6 [32%] subjects, 13 events; 235 mg: 9 [30%] subjects, 14 events; 259 mg: 2 [11%] subjects, 3 events; 294 mg: 1 [10%] subject, 1 event; 353 mg: 2 [22%] subjects, 3 events). No serious TEAEs and no TEAEs leading to death were reported, and no subjects withdrew from the study due to TEAEs.

**Table 2:** Summary of TEAEs in >1% of Subjects—Safety Analysis Set

	All TEAEs				Drug‐Related TEAEs			
	S + P 235 mg Fosnetupitant (N = 30)	Overall IV (N = 148)	Overall Oral (N = 134)	Total (N = 158)	S + P 235 mg Fosnetupitant (N = 30)	Overall IV (N = 148)	Overall Oral (N = 134)	Total (N = 158)
Subjects with ≥1 TEAE, *n* (%)	10 (33.3)	46 (31.1)	57 (42.5)	80 (50.6)	9 (30.0)	34 (23.0)	37 (27.6)	59 (37.3)
Number of TEAEs	20	83	86	169	14	55	53	108
MedDRA System Organ Class Preferred Term, *n* (%)								
Gastrointestinal disorders								
Abdominal pain	0 (0)	0 (0)	4 (3.0)	4 (2.5)	0 (0)	0 (0)	2 (1.5)	2 (1.3)
Abdominal pain upper	0 (0)	3 (2.0)	5 (3.7)	7 (4.4)	0 (0)	3 (2.0)	5 (3.7)	7 (4.4)
Constipation	0 (0)	2 (1.4)	10 (7.5)	12 (7.6)	0 (0)	2 (1.4)	10 (7.5)	12 (7.6)
Nausea	0 (0)	2 (1.4)	4 (3.0)	4 (2.5)	0 (0)	2 (1.4)	4 (3.0)	4 (2.5)
General disorders and administration‐site conditions								
Fatigue	0 (0)	6 (4.1)	3 (2.2)	9 (5.7)	0 (0)	6 (4.1)	3 (2.2)	9 (5.7)
Feeling hot	0 (0)	2 (1.4)	0 (0)	2 (1.3)	0 (0)	0 (0)	0 (0)	0 (0)
Infusion‐site thrombosis	4 (13.3)	8 (5.4)	2 (1.5)	10 (6.3)	4 (13.3)	8 (5.4)	2 (1.5)	10 (6.3)
Vessel puncture‐site thrombosis	5 (16.7)	14 (9.5)	14 (10.4)	25 (15.8)	0 (0)	0 (0)	0 (0)	0 (0)
Infections and infestations								
Nasopharyngitis	0 (0)	1 (0.7)	3 (2.2)	4 (2.5)	0 (0)	0 (0)	0 (0)	0 (0)
Musculoskeletal and connective tissue disorders								
Muscle spasms	0 (0)	1 (0.7)	1 (0.7)	2 (1.3)	0 (0)	0 (0)	0 (0)	0 (0)
Nervous system disorders								
Dizziness	2 (6.7)	3 (2.0)	5 (3.7)	8 (5.1)	2 (6.7)	3 (2.0)	3 (2.2)	6 (3.8)
Headache	2 (6.7)	12 (8.1)	21 (15.7)	28 (17.7)	2 (6.7)	12 (8.1)	17 (12.7)	25 (15.8)
Vascular disorders								
Phlebosclerosis	0 (0)	1 (0.7)	1 (0.7)	2 (1.3)	0 (0)	0 (0)	0 (0)	0 (0)

IV, infusion of 17.6 to 353 mg fosnetupitant and administration of placebo capsule; Oral, oral NEPA (netupitant 300 mg/palonosetron 0.5 mg) administration and placebo infusion; MedDRA, Medical Dictionary for Regulatory Activities; P, pilot crossover part; S, single‐ascending‐dose/single‐ascending‐dose crossover part; TEAE, treatment‐emergent adverse event.

The crossover study design was used for IV fosnetupitant 118‐ to 353‐mg dose cohorts, so most subjects received both IV fosnetupitant plus oral placebo (T) and oral NEPA plus IV placebo (R) in 2 distinct treatment periods with an adequate washout phase between them. No clinically significant differences in laboratory parameters, vital signs, or electrocardiograms were observed following the administration of T and R. The proportion of subjects with TEAEs was 31.1% when IV fosnetupitant was administered and 42.5% when oral NEPA was administered. The most commonly reported TEAEs (IV fosnetupitant vs oral NEPA) were headache in 17.7% of subjects (n = 28; 8.1% [n = 12] vs 15.7% [n = 21]), vessel puncture‐site thrombosis in 15.8% (n = 25; 9.5% [n = 14] vs 10.4% [n = 14]), and constipation in 7.6% (n = 12; 1.4% [n = 2] vs 7.5% [n = 10]). Doppler‐identified infusion‐site thrombosis events were reported in 6.3% (n = 10) of subjects, which included 5.4% (n = 8) of subjects following IV fosnetupitant and 1.5% (n = 2) following oral NEPA.

In total, 64% of TEAEs, occurring in 59 subjects, were considered study drug related by the investigator and DSMB (Table 2). TRAEs occurred in 23.0% (n = 34) and 27.6% (n = 37) of subjects following IV fosnetupitant and oral NEPA administration, respectively. The most common TRAEs (IV fosnetupitant vs oral NEPA) were headache in 15.8% of subjects (n = 25; 8.1% [n = 12] vs 12.7% [n = 17]) and constipation in 7.6% (n = 12; 1.4% [n = 2] vs 7.5% [n = 10]).

In total, 38 thrombotic events were reported through color Doppler examinations; of these, 28 involved the contralateral vein (arm), where blood sampling procedures were performed with no drug infusion, and 10 referred to the infusion vein (infusion arm). Eight of these 10 infusion‐site reactions were detected after IV fosnetupitant administration (from day 2 onward), while 2 occurred after IV placebo administration. All 10 reported cases of Doppler‐identified infusion‐site thrombosis were considered study drug related by the investigator and DSMB (5.4% [n = 8] of subjects following IV fosnetupitant and 1.5% [n = 2] following oral NEPA). All ISR events were assessed as mild in intensity, and all subjects recovered. None of the subjects presented symptoms such as swelling, or reported infusion‐site pain, suggesting the events were totally asymptomatic and only visible through Doppler examination.

### Pharmacokinetics

**Figure 2:**
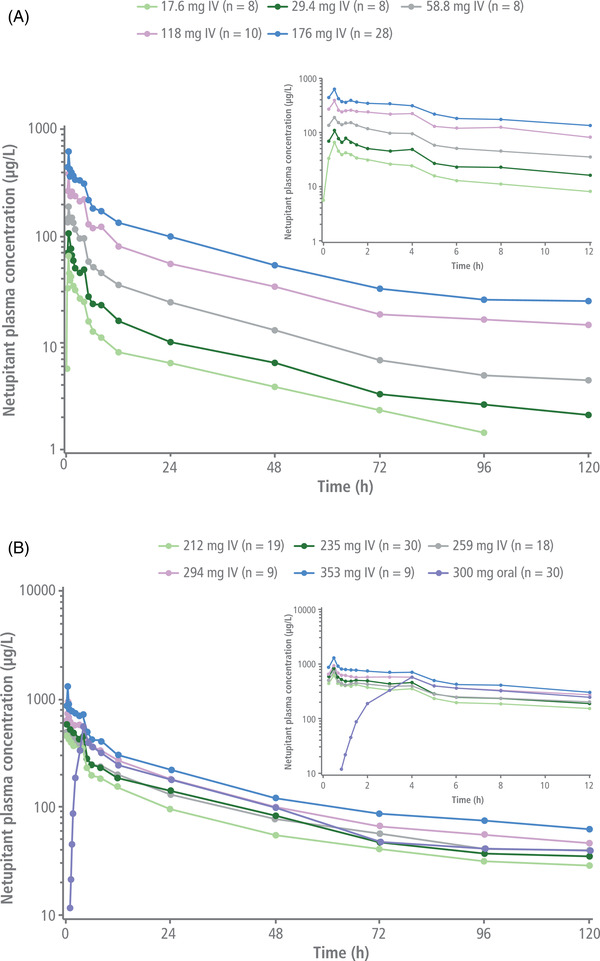
Mean plasma concentrations of netupitant over 12 and 120 hours after IV infusion of 17.6–176 mg fosnetupitant (A) and 212–353 mg fosnetupitant (SAD‐CO and P‐CO) or oral administration of netupitant 300 mg/palonosetron 0.5 mg (B). IV, intravenous; P‐CO, pilot crossover; SAD‐CO, single‐ascending‐dose crossover.

Plasma concentration–time profiles of netupitant following single IV administration of 17.6‐ to 353‐mg doses of fosnetupitant or oral NEPA are shown in Figure 2. Fosnetupitant was rapidly converted into netupitant after IV administration, resulting in limited mean systemic exposure to the prodrug. For all dose levels, the first quantifiable netupitant plasma concentrations appeared at 15 minutes after initiation of infusion and peaked at the end of the 30‐minute fosnetupitant infusion. Fosnetupitant C_max_ and AUC_0‐inf_ values increased dose proportionally. Netupitant C_max_ also increased dose proportionally, while AUC_0‐inf_ values showed slightly higher than dose‐proportional increases.

**Figure 3:**
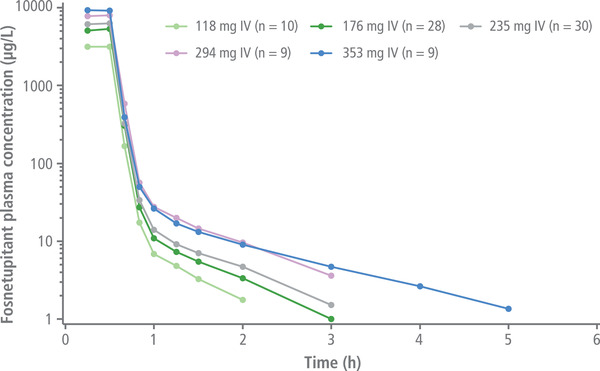
Mean plasma concentrations of fosnetupitant over 6 hours after IV infusion of 118–353‐mg fosnetupitant. IV, intravenous.

**Figure 4:**
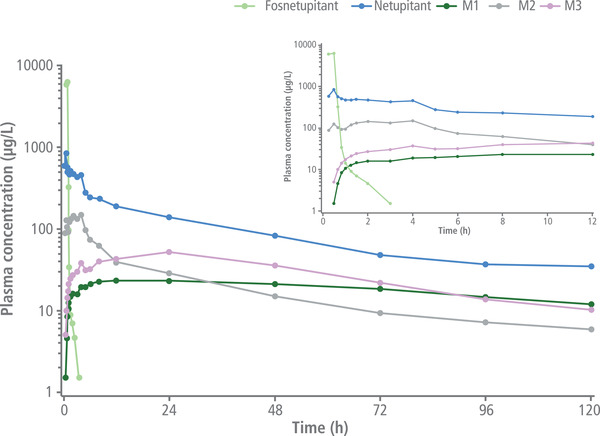
Mean plasma concentrations of fosnetupitant, netupitant, and netupitant metabolites M1, M2, and M3 over 12 and 120 hours after IV infusion of 235 mg fosnetupitant. IV, intravenous.

Following IV administration of 235 mg of fosnetupitant, the netupitant C_max_ was higher and reached earlier compared with oral netupitant 300 mg (Tables 3 and 4). The fosnetupitant concentration rapidly decreased (Figure 3) after the end of infusion. Thirty minutes after infusion, circulating fosnetupitant plasma concentrations dropped to <1% of mean C_max_. Netupitant elimination was slow, with a long mean terminal t_1/2_ of 36.05 hours (standard deviation, 6.81 hours) (Tables 3 and 4). Plasma concentrations of the metabolites M1, M2, and M3 were first detected within 1 hour after initiation of infusion, with M2 showing a higher concentration peak at an earlier time point compared with M1 and M3 (Figure 4).

**Table 3:** Pharmacokinetic Parameters (Mean ± SD) of Fosnetupitant, Netupitant, and Its Metabolites M1, M2, and M3 After IV Infusion of 235‐mg Dose of Fosnetupitant—PK‐R Analysis Set

Pharmacokinetic Parameter	Fosnetupitant (N = 30)	Netupitant (N = 30)	M1 (N = 30)	M2 (N = 30)	M3 (N = 30)
C_max_, μg/L	6431 ± 911.3	840.8 ± 172.6	26.1 ± 4.6	172.0 ± 78.7	54.1 ± 12.1
AUC_0‐last_, μg • h/L	2934 ± 362.3	12014 ± 2340	2326 ± 352.9	2583 ± 1254	3454 ± 739.9
AUC_0‐inf_, μg • h/L	2938 ± 362.1	13854 ± 2957	4070 ± 1667	2935 ± 1456	4313 ± 1183
t_max,_ h Median [min–max]	0.5 [0.3–0.5]	0.5 [0.5–4]	12 [2–48]	3 [0.5–4]	24 [4–48]
t_1/2_, h	0.956 ± 0.553	36.05 ± 6.812	89.34 ± 47.41	36.09 ± 24.77	47.32 ± 22.73

AUC_0‐inf_, area under the plasma concentration–time curve from time 0 to infinity; AUC_0‐last_, area under the plasma concentration–time curve from time 0 to the time of last measurable concentration; C_max_, maximum plasma concentration; IV, intravenous; M1, M2, and M3, main netupitant metabolites; Max, maximum; Min, minimum; P, pilot crossover part; PK‐R, restricted pharmacokinetic; S, single‐ascending‐dose/single‐ascending‐dose crossover part; SD, standard deviation; t_1/2_, elimination half‐life; t_max_, time to reach maximum concentration of drug in plasma.

**Table 4:** Pharmacokinetic Parameters (Mean ± SD) of Netupitant and Its Metabolites M1, M2, and M3 After Oral Dose of NEPA (300‐mg Netupitant/0.50‐mg Palonosetron)—PK‐R Analysis Set

Pharmacokinetic Parameter	Netupitant (N = 129)	M1 (N = 129)	M2 (N = 129)	M3 (N = 129)
C_max_, μg/L	477.3 ± 231.6	39.3 ± 14.0	213.9 ± 108.1	66.0 ± 22.2
AUC_0‐last_, μg • h/L	11317 ± 4278	3057 ± 879.5	2640 ± 1541	3783 ± 1303
AUC_0‐inf_, μg • h/L	13899 ± 5549	4819 ± 1847	2993 ± 1766	4620 ± 1845
t_max_, h Median [min–max]	4 [2–12]	8 [5–72]	4 [2–6]	12 [4–48]
t_1/2_, h	51.6 ± 30.9	76.9 ± 27.6	43.2 ± 50.0	46.4 ± 16.3

AUC_0‐inf_, area under the plasma concentration–time curve from time 0 to infinity; AUC_0‐last_, area under the plasma concentration–time curve from time 0 to the time of last measurable concentration; C_max_, maximum plasma concentration; M1, M2, and M3, main netupitant metabolites; Max, maximum; Min, minimum; P, pilot crossover part; PK‐R, restricted pharmacokinetic; S, single‐ascending‐dose/single‐ascending‐dose crossover part; SD, standard deviation; t_1/2_, elimination half‐life; t_max_, time to reach maximum concentration of drug in plasma.

The dose of 235‐mg fosnetupitant (T) was shown to be equivalent, in terms of netupitant systemic exposure, to the netupitant dose of 300 mg present in oral NEPA (R), on the basis of AUC_0‐last_ and AUC_0‐inf_ values. The 90%CI of the T/R AUC_0‐last_ and AUC_0‐inf_ GLSM ratio was within the 80.0%–125.0% interval for bioequivalence^37^ (Table 5).

**Table 5:** Bioequivalence Analysis of IV Fosnetupitant 235 mg (Test Formulation) and Oral Netupitant 300 mg (Reference Formulation)

Netupitant Parameter	Treatment	GLSM (Antilog)	Test (IV)/Reference (Oral) GLSM Ratio, %	90%CI (Lower–Upper Limits)
AUC_0‐last_, ng • h/mL	IV fosnetupitant (Test)	11876 (n = 30)	94.1	87.6–101.1
	Oral NEPA (Reference)	12621 (n = 30)		
AUC_0‐inf_, ng • h/mL	IV fosnetupitant (Test)	13290 (n = 26)	88.2	82.3–94.5
	Oral NEPA (Reference)	15073 (n = 21)		

AUC_0‐inf_, area under the plasma concentration–time curve from time 0 to infinity; AUC_0‐last_, area under the plasma concentration–time curve from time 0 to the time of last measurable concentration; GLSM, geometric least squares means; IV, intravenous.

For the bioequivalence assessment, the AUC_0‐inf_ values included in the analysis were only those with percentage extrapolation from t_last_ to infinity <20%.

## Discussion

IV formulations of NK_1_RAs, while effective in preventing CINV as part of a combination strategy (reviewed in Karthaus et al^14^), have the downside of causing ISRs, such as thrombophlebitis.^12^, ^13^, ^14^ Conversely, IV 5‐HT_3_RAs are rarely associated with ISRs, although hypersensitivity reactions have been observed following ondansetron and tropisetron infusions^38^, ^39^; these are related to the drug formulations rather than to the agents themselves. The difference in the ISR occurrence between the 2 drug classes reflects their different pharmacodynamic properties and the innate chemical and physical properties of NK_1_RAs, such as their amphiphilic nature and low solubility.^15^, ^16^, ^17^, ^18^ The relatively high incidence of ISRs has driven the development of improved IV formulations of NK_1_RAs.^22^

The current study aimed to evaluate the safety and PK of increasing doses of the IV fosnetupitant prodrug in healthy volunteers. Following IV administration, fosnetupitant was converted in vivo to netupitant. A 30‐minute IV infusion of a single dose of fosnetupitant between 17.6 and 353 mg (<235 mg in 89 subjects; 235 mg in 30; >235 mg in 39) had a manageable safety profile that was associated with occurrence of AEs of only mild or moderate intensity. Furthermore, the overall number of TEAEs and the number of subjects experiencing TEAEs did not increase with rising doses of fosnetupitant. ISR thrombotic events in this study were evaluated with color Doppler ultrasound scanning of the infusion and contralateral veins. Doppler ultrasound scanning is a highly sensitive technique that is not commonly used in clinical practice or in clinical trials for assessing blood vessel damage following IV injection. In total, 38 thrombotic events were identified using this technique on both the infusion arm veins following IV administration of fosnetupitant or placebo and the contralateral arm veins following blood sampling procedures. Of these, only 10 transient and mild thrombotic events were reported in the infusion arm and considered treatment related. Eight of these 10 cases occurred when subjects received IV fosnetupitant, with all events occurring from day 2 onward, and 2 were reported following administration of the IV placebo solution. All ISRs were of mild intensity, and all subjects recovered. The fact that none of the ISRs were associated with symptoms such as swelling or pain suggests these events were asymptomatic and only perceptible through Doppler. Taken together, these observations may suggest that the thromboses observed in our study likely originated from mechanical perturbations associated with the injection procedure rather than as a result of the fosnetupitant formulation. Unfortunately, no other studies have employed color Doppler ultrasound to determine all levels of thrombotic events, so no interstudy comparisons can be made.

Our results are consistent with those reported from a phase 1 clinical trial in patients receiving cisplatin and IV NEPA (n = 24, single dose),^25^ a phase 3 safety study of IV NEPA in patients receiving HEC (n = 203, 667 cycles total),^26^ and a phase 3b trial in patients who received AC and IV NEPA,^27^ where no IV NEPA‐related ISRs were observed. The latter study is of particular interest, as it enrolled patients who were receiving AC‐based chemotherapy, a regimen that is associated with increased risk of ISRs, with rates of 7%–11% previously reported.^40^, ^41^, ^42^, ^43^ In this trial, 200 patients were administered IV NEPA for a total of over 600 cycles of AC‐based chemotherapy. The majority of patients received the drug via the peripheral line, and no treatment‐emergent ISRs were observed during the study.^27^ The safety profile of oral NEPA has been well established in several phase 2 and 3 clinical trials that have enrolled over 1500 patients with cancer.^44^ The safety data obtained herein from 158 healthy volunteers who received IV fosnetupitant, of whom 30 received the 235‐mg dose present in IV NEPA, are similar to that reported for oral NEPA during its clinical development.

In contrast to our study, data from the pivotal trials of the prodrug fosaprepitant reported the occurrence of ISRs. In one phase 3 trial, the incidence of ISRs was 2.2% in 1143 patients receiving IV fosaprepitant compared with 0.4% of 1169 patients receiving oral aprepitant, both administered in combination with 5‐HT_3_RA–dexamethasone for cisplatin‐based chemotherapy. Severe ISRs associated with either regimen were pain and thrombophlebitis, with the latter occurring more frequently with IV fosaprepitant.^45^ Another trial evaluated IV fosaprepitant versus IV placebo in combination with 5‐HT_3_RA–dexamethasone in patients receiving non‐AC moderately emetogenic chemotherapy^23^; infusion‐site thrombophlebitis was reported in 0.6% of 504 patients receiving IV fosaprepitant compared with no cases reported among the 497 patients receiving IV placebo. Retrospective studies have reported even higher incidences of ISRs with IV fosaprepitant. In total, 25% of 127 patients who received IV fosaprepitant in conjunction with AC‐based chemotherapy experienced at least 1 ISR, and 13% had hypersensitivity systemic reactions, which primarily involved edema/swelling, erythema, or dermatitis; no patients experienced anaphylaxis.^11^ Another study, which included 98 patients with breast cancer who received IV fosaprepitant and AC chemotherapy, reported an ISR incidence of 34.7% versus only 2.3% among 44 patients receiving oral aprepitant.^13^

Potential explanations for the disparity in the incidence of ISRs between IV fosaprepitant and IV fosnetupitant may be the presence of intrinsic factors regarding the structure of the 2 prodrug molecules and, possibly, the difference in local concentrations of the parent drugs (aprepitant and netupitant), as high concentrations of components that are damaging or irritating to the cell membrane may cause phlebitis^10^ and a higher incidence of ISRs. A possible reason for the low incidence of ISRs with fosnetupitant may involve the intrinsic nonirritant, non–coagulation‐inducing properties of the prodrug and the limited concentration of unbound netupitant at the local infusion site due to the high plasma protein binding of netupitant (netupitant unbound fraction in human plasma is <0.5%), which may preclude damage to vascular endothelial cells. Additional potential explanations may relate to the absence of surfactants and other excipients, present in other products, that increase the risk of allergic and hypersensitivity reactions.

The aprepitant emulsion HTX‐019, containing egg lecithin, ethanol, sodium oleate, soybean oil, sucrose, and water for injection, is associated with fewer ISRs than fosaprepitant. A comparison of IV HTX‐019 and IV fosaprepitant in healthy subjects showed 2% of 196 subjects who received HTX‐019 and 10% of 200 patients who received fosaprepitant reported ISRs.^7^ However, as the AEs were monitored for only 60 minutes, events that occurred later would not have been captured in this study. A retrospective study of IV HTX‐019 evaluated the safety of the drug following a 2‐minute injection in 600 patients with cancer who received moderately emetogenic chemotherapy or HEC.^46^ There were no reports of ISRs in this study, although again, safety monitoring was undertaken for only 60 minutes after injection, so the data should be interpreted with some caution. In addition, the majority of patients (76%) had a central IV line, which may decrease the likelihood of ISRs compared with a peripheral line. Finally, another retrospective study evaluated the safety profile of IV HTX‐019 after a 30‐minute infusion in 147 patients; no ISRs were reported, but here also patients were monitored for only 1 hour following HTX‐019 administration.^47^

Because fosnetupitant is rapidly and nearly completely transformed to netupitant, IV 235‐mg fosnetupitant is expected to follow PK similar to oral 300‐mg netupitant, except for differences intrinsic to the different administration routes (absorption). Similar to PK studies of IV NEPA in patients with cancer,^24^ in the present study fosnetupitant C_max_ was reached at the end of the 30‐minute infusion, with <1% of the prodrug being detectable 30 minutes after the end of infusion. Small disparities in PK parameters between the 2 studies could be explained by differences at distribution, metabolism, and elimination levels between healthy subjects and patients with cancer. As observed for oral netupitant, fosnetupitant IV infusion leads to formation of M1, M2, and M3 metabolites, of which M3 is the most active. In the present study, M3 total exposure following fosnetupitant infusion was ≈30% of that of netupitant; therefore, it is expected that M3 may contribute substantially to the overall inhibition of NK_1_ receptors by fosnetupitant. On the other hand, the less abundant and less active M1 and M2 are expected to play a marginal role in overall antiemetic activity. Regarding potential drug‐drug interactions, the fosnetupitant 235‐mg dose is expected to have an inhibitory effect of CYP3A4 similar to that observed with oral netupitant 300 mg.^28^ As such, in clinical studies IV NEPA was coadministered with the same reduced dose of dexamethasone used in oral NEPA regimens.^25^, ^26^, ^27^ Importantly, no additional safety issues related to dexamethasone were reported.^25^, ^26^, ^27^ Regarding pharmacodynamics, NK_1_ receptor occupancy in the brain may be expected to occur faster with IV fosnetupitant 235 mg than with oral netupitant 300 mg, because netupitant C_max_ is reached ≈3.5 hours earlier with the IV formulation. As the dynamics of netupitant concentration are similar from 4 (time to reach maximum concentration in plasma following oral netupitant 300 mg) to 120 hours after administration, the duration of NK_1_ receptor occupancy and its clinical effects are expected to be similar to fosnetupitant 235‐mg dose, as previously observed with netupitant 300 mg.^29^ However, these predictions still need to be confirmed in the clinic.

The current study established that a single IV dose of 235‐mg fosnetupitant is equivalent, in terms of netupitant exposure, to the 300‐mg dose of netupitant present within the oral NEPA formulation. The similarity in systemic exposures of netupitant suggests that NK_1_ receptor occupancy is comparable after IV fosnetupitant and oral NEPA administration. In turn, this may translate into similar antiemetic efficacy of IV fosnetupitant 235 mg compared with oral netupitant 300 mg (as a component of NEPA).

## Conclusions

A single infusion of 235 mg IV fosnetupitant presented a manageable safety profile that is in line with the favorable safety profile previously observed in phase 2/3 trials with oral NEPA. The lack of ISRs may be due to the intrinsic chemical characteristics of fosnetupitant, thereby allowing a formulation containing no surfactants, excipients, or components associated with allergies. This may contribute to its tolerability and potentially represent an advantageous treatment alternative, in particular for patients who are at risk of allergic reactions to components present in other NK_1_RA IV formulations. In addition, because amphiphilic compounds can be damaging to the cell membrane at high concentrations,^10^ possible low concentration of the active moiety at the infusion site could result in good local tolerability.

The IV fosnetupitant 235‐mg dose was shown to be equivalent, in terms of netupitant systemic exposure, to the 300‐mg dose of netupitant present in oral NEPA. These data suggest that NEPA, which is the only fixed‐dose combination agent and the only injectable combination for which the compatibility of the NK_1_ and 5‐HT_3_RA have been assessed in the same formulation, may be considered a convenient antiemetic combination. The agent simultaneously targets the 2 main emetic pathways through the combination of the pharmacologically and clinically different 5‐HT_3_RA palonosetron with the NK_1_RA (fos)netupitant, which has proven safe and tolerable. A single dose offers simple and convenient CINV prophylaxis during the 5‐day emetic risk period following chemotherapy, and thus should contribute to reduced potential administration errors and increased compliance through its ease of use.

## Conflicts of Interest

Timothy Tyler: speaker for MorphoSys/Incyte, G1 Therapeutics, Alexion, Sanofi Genzyme, Pfizer Biosimilars, Genentech, Amgen/Onyx, and BMS; consultant for TerSera, Sandoz, and Secura Bio; advisory board member for Corvida Medical, C.S. Lewis Foundation. Armin Schultz: CRS Mannheim GmbH employee. Alessio Venturini: Former Helsinn employee. Claudio Giuliano: Helsinn employee. Alberto Bernareggi: Helsinn employee. Riccardo Spezia: Helsinn employee at the time of study conduct and current freelance consultant for Helsinn. Daniel Voisin: Helsinn employee. Valentino Stella: consultant for Helsinn. Helsinn Healthcare SA participated in the design of the study; collection, analysis, and interpretation of data; approval of the manuscript; and the decision to submit the article for publication

## Supporting information

Supplemental Information
