# Supplementary material for: Challenges in the Development of Intravenous Neurokinin‐1 Receptor Antagonists: Results of a Safety and Pharmacokinetics Dose‐Finding, Phase 1 Study of Intravenous Fosnetupitant
Source: Clin Pharmacol Drug Dev. 2022 Oct 20;11(12):1405–18. doi: 10.1002/cpdd.1183 (PMC10092591; doi:10.1002/cpdd.1183)

**Supplementary Material**

Tyler T, Schultz A, Venturini A, et al. Challenges in the Development of Intravenous Neurokinin-1 Receptor Antagonists: Results of a Safety and Pharmacokinetics Dose-Finding, Phase 1 Study of Intravenous Fosnetupitant. *Clin Pharmacol Drug Dev*.

**Supplementary Methods.** Analytic methods.

**Supplementary Figure.** Subject disposition.

**Supplementary Methods**

*Analytic methods*

Fosnetupitant in human K3-EDTA plasma was quantified by a sensitive and specific internally standardized liquid chromatography-tandem mass spectrometry (LC-MS/MS) method in the positive electrospray ionization (ESI) mode with multiple reaction monitoring (MRM), using repaglinide as the internal standard (IS). For sample preparation, extraction was performed by protein precipitation. Plasma samples (50 μL) were mixed with the IS solution (150 μL), and the supernatant (100 μL) was transferred into a 96-well plate. A 2.5-µL aliquot was injected into the LC-MS/MS for analysis. Chromatographic separation using quaternary high-performance liquid chromatography (HPLC) pump (Flux Instruments Rheos Allegro, Burladingen, Germany) was performed on a Hydro-RP 4-μm Synergi™ 80 Å column (inner diameter 2 mm, length 50 mm; Phenomenex Helvetia GmbH, Basel, Switzerland) with a Security Guard™ Cartridge C8 pre-column (inner diameter 3 mm, length 4 mm; Phenomenex Helvetia GmbH). The mobile phase consisted of water (A) and acetonitrile (B) containing 0.1% formic acid. Detection was performed with API 4000™ or 5000™ LC-MS/MS Systems (AB Sciex LLC, Framingham, MA, USA). The analytic gradient conditions were from 80% A : 20% B to 5% A : 95% B in 2.1 minutes, followed by elution under isocratic conditions for up to 5.2 minutes. The flow rate was 500 µL/minute. The MS parameters were: ESI-positive MRM; ion source: Turbo V^TM^ Ion Source (AB Sciex LLC); curtain gas flow: nitrogen 20.00 psi; ion source gas 1 and 2: dried air 60 and 40 psi; collision gas: nitrogen, collision energy 35 eV. Mass transitions (m/z): 689.60 → 579.40 (fosnetupitant), 453.40 → 230.30 (IS). Typical retention time: 1.5 minutes (fosnetupitant), 1.3 minutes (IS). Validation was performed according to the US Food and Drug Administration (FDA) Guidance for Industry on Bioanalytical Method Validation (2001) and showed that the method was suitable for analysis of fosnetupitant in human plasma, having good overall performance in the calibration range from 2.00 (lower limit of quantitation [LLOQ]) to 1000 ng/mL (upper limit of quantitation [ULOQ]). In this concentration range, the within-run accuracy and precision ranged from -8.4% to 8.5% and from 0.8% to 9.9%, respectively, and the between-run accuracy and precision ranged from -4.6% to 0.6% and from 3.1% to 7.8%, respectively.

Netupitant, M1, M2, and M3 were determined in human K3-EDTA plasma by a sensitive and specific internally standardized LC-MS/MS method in the positive ESI mode. ISs were netupitant-D6 for netupitant, RO 0681133-002-001 for M1, RO 0713001-001-001 for M2, and RO 0731519-001-001 for M3. Before detection in MRM by MS, analytes and their ISs were separated from endogenous compounds on an analytic narrow-bore column. Extraction of plasma samples was performed in 96-well solid liquid extraction (SLE) plate Chem Elut SLE (Agilent Technologies [Schweiz] AG, Basel, Switzerland). Plasma samples (80 μL) were diluted with IS (20 μL) solution and buffer pH 9.0 (80 μL). Then samples were pipetted into a 96-well plate and vortexed at maximum speed for 5 seconds. For extraction, the 180-μL samples were transferred into the collection plate, allowing them to equilibrate for 10 minutes. Thereafter, the analytes were eluted four times with 200 μL of the extraction (70% diethyl ether, 30% ethyl acetate) solution per well each time. The SLE plate was evaporated to dryness under a stream of nitrogen at 55°C. Dried extracts were reconstituted with 300 μL of the flux (A: 2.5 L acetonitrile, B: 0.4 L acetonitrile + 1.6 L water + 1.82 g ammonium acetate; 1:1), vortexed at maximum speed for 5 seconds, and centrifuged at 2500 g. A 5-µL aliquot was injected into the LC-MS/MS for analysis. Chromatographic separation was performed using the Flux Rheos 2200 or Flux Rheos Allegro (Flux Instruments Rheos Allegro) HPLC pump on the Synergi 4-μm Hydro-RP 80 Ǻ (inner diameter 2.0 mm, length 50 mm; Phenomenex Helvetia GmbH) column and C8 Security Guard Cartridge (inner diameter 2 mm, length 4 mm) pre-column. Detection was performed with API 5000™ LC-MS/MS System (AB Sciex LLC). The mobile phase A was acetonitrile and the mobile phase B was 1.6 L water + 0.4 L acetonitrile + 1.82 g ammonium acetate. The analytic gradient conditions were from 50% A : 50% B to 95% A : 5% B in 1.5 minutes, followed by elution under isocratic conditions for up to 2.5 minutes. The flow rate was 650 µL/minute. MS parameters were: ESI positive with MRM; ion source: Turbo V Source; curtain gas flow: nitrogen 15 psi; ion source gas 1 and 2: dried air 50 and 70 psi; collision gas: nitrogen; collision energy 31 eV netupitant and 41 eV netupitant IS, 43 eV M1 and M1 IS, 36 eV M2 and M2 IS, 31 eV M3 and 41 eV M3 IS. Mass transitions (m/z): 579.4 → 522.4 (netupitant), 585.4 → 528.4 (netupitant IS), 565.3 → 522.3 (M1), 571.3 → 528.3 (M1 IS), 595.4 → 508.4 (M2), 601.4 → 514.4 (M2 IS), 595.4 → 520.4 (M3), 601.4 → 526.4 (M3 IS). Typical retention times were 2.2 minutes for netupitant and netupitant IS, 1.9 minutes for M1 and M1 IS, 1.7 minutes for M2 and M2 IS, 1.3 minutes for M3 and M3 IS. Validation was performed according to the FDA Guidance for Industry on Bioanalytical Method Validation (2001) and showed that the method was suitable for analysis of netupitant, M1, M2, and M3 in human plasma, having good overall performance in the set calibration range from 2.00 (LLOQ) to 500 ng/mL (ULOQ). In this concentration range, the within-run accuracy and precision ranged for netupitant from -1.58% to 3.56% and from 1.45% to 4.88%, respectively; for M1 from -7.16% to 1.33% and from 1.51% to 3.86%, respectively; for M2 from -6.44% to -1.61% and from 1.29% to 4.75%, respectively; for M3 from -3.05% to -0.13% and from 1.54% to 5.00%, respectively. The between-run accuracy and precision ranged for netupitant from 0.41% to 1.92% and from 2.92% to 3.62%, respectively; for M1 from -4.08% to -2.18% and from 3.07% to 3.18%, respectively; for M2 from -5.09% to -2.99% and from 3.06% to 3.70%, respectively; for M3 from -2.52% to -0.97% and from 2.39% to 2.85%, respectively.

**Supplementary Figure.** Subject disposition. F-CO, final crossover; IV, intravenous infusion of 17.6 to 353 mg fosnetupitant and administration of placebo capsule; N, number of subjects; P-CO, pilot crossover; PO, oral NEPA (netupitant 300 mg/palonosetron 0.5 mg) fixed-dose administration and placebo infusion; SAD, single ascending dose; SAD-CO, single ascending dose crossover.


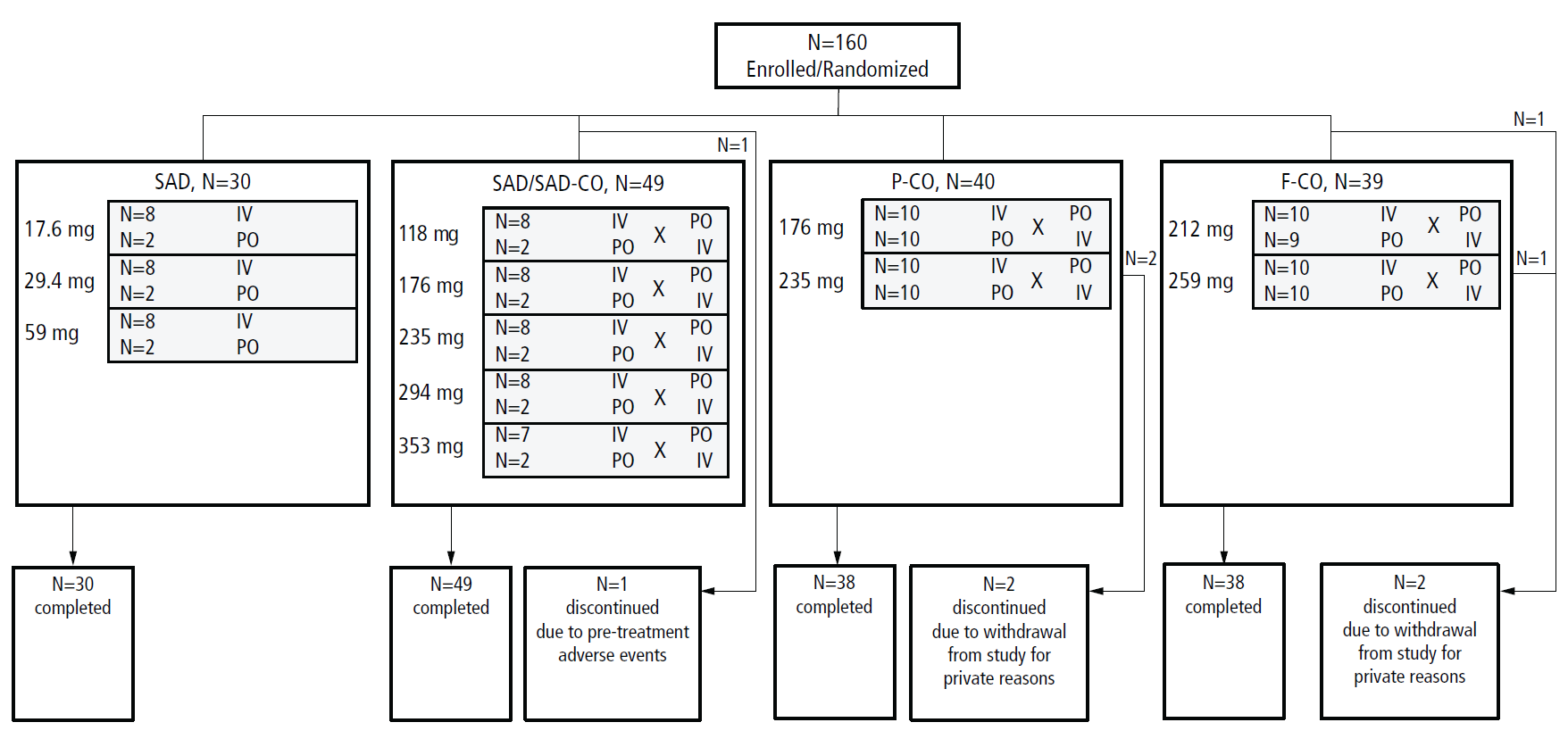

Supplement: Supplementary file 1 — Supplemental Information [file CPDD-11-1405-s001.docx]
